# Retinoic acid-loaded nanoparticles enhance macrophage-mediated control of *Leishmania infantum*

**DOI:** 10.55730/1300-0152.2803

**Published:** 2026-03-26

**Authors:** Zeynep İŞLEK KÖKLÜ, Merve GÜNDOĞDU, Zeynep Dila YÜKSEL

**Affiliations:** Department of Genetics and Bioengineering, Faculty of Engineering and Natural Sciences, Yeditepe University, İstanbul, Turkiye

**Keywords:** All-trans retinoic acid (ATRA), leishmaniasis, solid lipid nanoparticles (SLNs), *Leishmania infantum*, macrophages

## Abstract

**Background/aim:**

Visceral leishmaniasis, primarily caused by *Leishmania (L.) infantum*, remains a major global health challenge due to limitations in current chemotherapeutic options, including toxicity and emerging drug resistance. Host-directed therapeutic approaches are increasingly recognized as promising alternatives. All-trans retinoic acid (ATRA) is an immunomodulatory molecule with host-dependent effects on macrophage function; however, its therapeutic use is hindered by instability and poor solubility. Solid lipid nanoparticles (SLNs) offer a controlled and biocompatible delivery platform capable of enhancing intracellular drug accumulation.

**Materials and methods:**

ATRA-loaded SLNs were prepared and characterized for size, polydispersity index, zeta potential, and morphology. Their antileishmanial activity was evaluated against extracellular *L. infantum* promastigotes, noninfected RAW 264.7 macrophages, and *L. infantum*-infected macrophages using resazurin-based assays and xCELLigence real-time cell analysis.

**Results:**

Neither free ATRA nor ATRA-loaded SLNs exhibited significant inhibitory activity against extracellular promastigotes at concentrations up to 75 μM. In contrast, both forms of ATRA demonstrated marked dose-dependent inhibition in infected macrophages, with a significantly enhanced intracellular response observed in the SLN formulation, while maintaining excellent biocompatibility in noninfected macrophages. Enhanced uptake and sustained intracellular release are likely contributors to the improved efficacy of the SLN system.

**Conclusion:**

The findings reveal that ATRA exerts its antileishmanial activity primarily through host-dependent mechanisms that become apparent within infected macrophages, and that encapsulation into SLNs markedly amplifies this intracellular effect while preserving cell viability. ATRA-loaded SLNs thus represent a promising host-directed therapeutic strategy for the treatment of visceral leishmaniasis.

## 1. Introduction

Leishmaniasis remains one of the most challenging neglected tropical diseases, affecting millions of people worldwide and causing substantial morbidity and mortality, particularly in regions where *Leishmania infantum* and *L. donovani* drive visceral leishmaniasis (VL) (WHO, 2013; Arnold et al., 2022). The intracellular nature of the parasite, its capacity to remodel host macrophage metabolism, and the limited efficacy and toxicity concerns associated with current antileishmanial drugs highlight the urgent need for alternative and host-compatible therapeutic strategies (Croft et al., 2006; Sundar and Chatterjee, 2006; Sundar and Singh, 2016). As macrophages serve as both the primary host cell and the central effector of parasite control, therapeutic approaches that modulate macrophage function or alter the intracellular environment represent a promising direction for VL drug development.

All-trans retinoic acid (ATRA), the biologically active metabolite of vitamin A, has gained increasing attention for its immunomodulatory effects in infectious diseases. ATRA regulates a wide range of cellular processes, including differentiation, lipid metabolism, antigen presentation, and cytokine production, through retinoic acid receptors (RAR/RXR) and retinoid-dependent transcriptional networks (Freemantle et al., 2003; Bidad et al., 2011; Liu et al., 2015; Bahlool et al., 2022). In the context of leishmaniasis, however, its role remains complex and, at times, contradictory. In some studies it is reported that ATRA enhances susceptibility to *L. major* by favoring an M2-like macrophage phenotype, whereas in others it is demonstrated that ATRA restores cholesterol homeostasis and improves parasite clearance in *L. donovani*-infected macrophages (Prakash and Rai, 2022, 2023). Clinical and ex vivo data from VL patients further suggest that ATRA modulates IL-10, TGF-β, and IL-17 production in a context-dependent manner (Maciel et al., 2014; Vellozo et al., 2017), acting not purely as an antiparasitic agent but as a regulator of host immune pathways. These observations collectively point toward a dual and highly environment-dependent role of ATRA in host–parasite interactions.

Despite its immunological potential, the therapeutic use of ATRA is limited by poor solubility, instability, rapid metabolism, and dose-limiting toxicity. Nanotechnology-based delivery systems, particularly solid lipid nanoparticles (SLNs), offer a means to overcome these obstacles by improving drug stability, enhancing intracellular accumulation, and enabling controlled release (Zeynep Islek et al., 2024). SLNs within the 100–150 nm size range are readily internalized by macrophages and can effectively deliver bioactive molecules to intracellular compartments where *Leishmania* resides. Previous studies have shown that nanoencapsulated retinoids can modulate immune responses safely, yet their potential for direct intracellular antileishmanial applications remains insufficiently explored (Bezerra et al., 2019).

Considering the immunomodulatory properties of ATRA and the need for effective host-directed therapeutic strategies in VL, the evaluation of SLN-based delivery systems may provide important insights into the use of retinoids to enhance macrophage-mediated control of amastigotes. Addressing the limitation in drug toxicity, in the present study the antileishmanial activity of free ATRA versus ATRA-loaded SLNs was investigated using promastigotes, infected macrophages, and noninfected RAW 264.7 macrophages. By integrating nanoparticle characterization with biological assays, the aim was to elucidate the mechanism of ATRA-mediated antileishmanial activity and to evaluate how SLN encapsulation enhances its intracellular efficacy while preserving macrophage viability.

## 2. Materials and methods

Empty and ATRA-loaded SLNs were produced using a modified version of the previously reported film hydration approach (Zeynep Islek et al., 2024). In brief, ATRA was combined with the lipid components (DPPC, tripalmitin, and cholesterol) dissolved in 2 mL of chloroform at a ratio of 10:1:0.3 (mg). The total phospholipid-to-tripalmitin ratio was maintained between 0.1 and 0.15 (w/w) throughout preparation. Following complete removal of the solvent, the resulting thin lipid film was hydrated with an aqueous phase. The dispersion was then subjected to ultrasonication at 65 °C for 50 min to obtain a homogeneous nanoparticle suspension.

### 2.1. Characterization of SLNs

#### 2.1.1. Particle size, polydispersity, and zeta potential

The mean hydrodynamic diameter, polydispersity index (PDI), and zeta potential of ATRA-loaded SLNs were measured using dynamic light scattering (DLS) on a Zetasizer Nano ZS (Malvern Instruments, Malvern, Worcestershire, UK) at 25 °C. Samples were diluted 1:10 in distilled water to ensure optimal scattering intensity. Measurements were performed in triplicate and results were expressed as mean ± SD.

#### 2.1.2. Morphological analysis by transmission electron microscopy (TEM)

Morphological features of SLNs were analyzed using a JEM-2100 Plus transmission electron microscope. ATRA-loaded SLNs were diluted 1:60 in distilled water and a droplet was placed onto formvar/carbon-coated copper grids. After overnight adsorption, the grids were stained with 2% uranyl acetate for 1 min, blotted gently, and dried at room temperature. Images were acquired at an accelerating voltage of 200 kV.

#### 2.1.3. Quantification of encapsulated ATRA

ATRA concentration within SLNs was determined based on its intrinsic fluorescence. Samples were excited at 352 nm and emission spectra were recorded using a Spectramax i3 Multi-Mode Microplate Reader. ATRA stock solution (1 mg/mL) was serially diluted to generate standard concentrations (0–100 μg/mL), and SLN samples were diluted 1:10 before measurement. A standard calibration curve was used to calculate ATRA content in the SLN formulations.

#### 2.1.4. Encapsulation efficiency

Encapsulation efficiency (EE%) was calculated using the following equation:


$$\text{EE }(\%)=({\text{W}}_{\text{encapsulated}}/{\text{W}}_{\text{total}})\times 100,$$

where *W_encapsulated_* represents the amount of ATRA detected in the SLNs after centrifugation and *W_total_* represents the total amount of ATRA initially used in the formulation.

### 2.2. Parasite and macrophage cell culture

#### 2.2.1. Culture of *L. infantum* promastigotes

*L. infantum* promastigotes were cultured at 25 °C in RPMI 1640 GlutaMAX supplemented with 10% iFBS, 50 U/mL penicillin, 50 μg/mL streptomycin, and 20 mM HEPES (pH 7.4). Infective metacyclic forms were obtained from stationary-phase cultures, which were not passaged beyond seven generations to maintain infectivity. Parasites were harvested by centrifugation at 3000 × *g* for 10 min.

#### 2.2.2. Culture of RAW 264.7 macrophages

RAW 264.7 murine macrophages were maintained at 37 °C in a humidified 5% CO_2_ atmosphere in Dulbecco’s modified Eagle’s medium supplemented with 10% FBS, 2 mM L-glutamine, 100 U/mL penicillin, and 100 μg/mL streptomycin. Cells were subcultured every 3 days to maintain exponential growth.

#### 2.2.3. Determination of antileishmanial activity of the SLN formulation on *L. infantum* promastigotes

The effects of free ATRA and ATRA-loaded SLNs on promastigote viability were assessed using the resazurin assay. Exponential-phase promastigotes were seeded in 96-well plates (3 × 10^5^ parasites/well) and treated with 0–75 μM free ATRA or ATRA-loaded SLNs. After 24, 48, and 72 h of incubation at 25 °C, 10% of a 2.5 mM resazurin solution was added, and fluorescence was measured (Ex: 560 nm/Em: 590 nm). Viability (%) was calculated relative to untreated controls.

#### 2.2.4. Cytotoxicity assay on RAW 264.7 macrophages

RAW 264.7 cells were seeded in 96-well plates (1 × 10^4^ cells/well) and treated with various concentrations (0–75 μM) of free ATRA and ATRA-loaded SLNs for 24, 48, and 72 h. Following incubation, cells were treated with 10% resazurin for 4 h and fluorescence was measured at 560/590 nm. Cell viability was expressed relative to untreated controls.

#### 2.2.5. xCELLigence real-time cell analysis (RTCA) for *Leishmania*-infected macrophages

RAW 264.7 macrophages were seeded into E-plates of the xCELLigence RTCA system (Agilent Technologies) at a density of 8000 cells per well and allowed to adhere for 24 h. Subsequently, macrophages were infected with *Leishmania infantum* promastigotes at a parasite-to-cell ratio of 10:1. After 3 h of incubation, noninternalized parasites were removed by washing with PBS and fresh medium was added. Infected macrophages were then treated with free ATRA or ATRA-loaded SLNs at concentrations ranging from 0 to 75 μM. Cell behavior was continuously monitored for 72 h using the xCELLigence RTCA system. The system measures electrical impedance generated by adherent cells on microelectrodes integrated into the bottom of the wells, which is expressed as the cell index (CI). Changes in CI reflect alterations in macrophage adhesion, morphology, and proliferation over time. Real-time CI curves were recorded, and the growth kinetics of treated cells was compared with that of untreated infected macrophage controls to evaluate treatment-associated changes in macrophage proliferation and viability.

### 2.3. Statistical analysis

GraphPad Prism Software (version 8.0.1) was used to perform the statistical analysis. Error bars represent the standard error of the mean. The datasets were compared using one-way and two-way ANOVA, followed by Tukey’s or Šidák multiple comparison test. Differences were considered statistically significant at (*) p ≤ 0.05, (**) p ≤ 0.01, (***) p ≤ 0.001, and (****) p ≤ 0.0001.

## 3. Results

### 3.1. Characterization of SLN formulations

#### 3.1.1. Measurement of size, polydispersity, and zeta potential and morphological analysis by TEM

**Figure 1:**
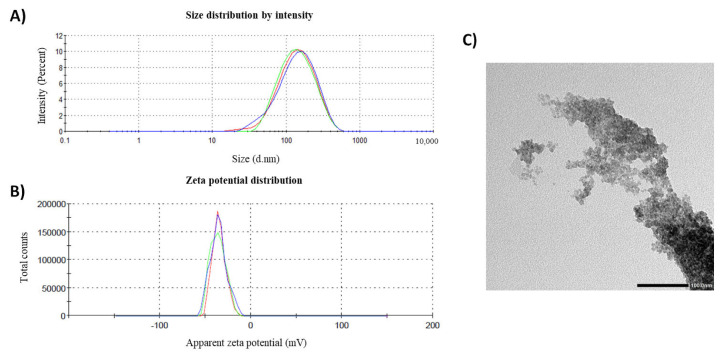
The representative graph of (A) average particle size, (B) average zeta potential values, and (C) morphological TEM analysis of ATRA-loaded SLNs (scale bar: 100 nm).

At least three independently prepared ATRA-loaded SLN formulations were evaluated for their particle size, PDI, and zeta potential. The average hydrodynamic diameter of the ATRA-loaded SLNs was 120.6 ± 10.8 nm, with a PDI of 0.242 ± 0.013, indicating a moderately narrow and uniform size distribution (Figure 1A). The conductivity of the dispersion was consistent with values expected for lipid-based colloidal systems.

All formulations exhibited a distinctly negative surface charge (Figure 1B), with ATRA-loaded SLNs presenting a zeta potential of −35.1 ± 7.20 mV. The relatively high negative surface charge indicates that the formulation helps prevent the particles from coming together, a feature typically associated with improved colloidal stability both during storage and in biological fluids. Although slight batch-to-batch variations were noted, the overall zeta potential values did not differ significantly (p ≥ 0.05), suggesting that the preparation method reliably produces consistent surface properties.

TEM provided further support for the physicochemical findings. The SLNs appeared mostly spherical, with occasional minor irregularities, yet the particles remained well separated, and a few minor particle contacts were observed, consistent with normal drying effects during grid preparation, but no significant aggregation or fusion was detected (Figure 1C). The absence of aggregation in the micrographs is consistent with the DLS results and provides additional confirmation that the nanoparticles produced are structurally stable and exhibit a uniform dispersion profile.

#### 3.1.2. Determination of ATRA content in SLN

The ATRA concentration in the SLN formulation was 59 μg/mL (197 μM).

#### 3.1.3. Encapsulation efficiency

After the removal of the unincorporated ATRA from the system via centrifugation, encapsulation efficiencies of the ATRA-loaded SLNs were 19.6%.

### 3.2. Cell culture studies

#### 3.2.1. Antileishmanial effect of the ATRA and ATRA-loaded SLNs on the growth of *L. infantum* promastigotes

**Figure 2:**
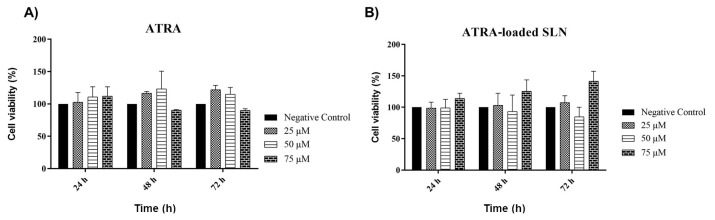
The effect of free ATRA and ATRA-loaded SLNs on *L. infantum* promastigotes. Cell viability analysis of *L. infantum* treated with (A) free ATRA (in ethanol) and (B) ATRA-loaded SLNs was investigated at various concentrations (0–75 μM) for 24, 48, and 72 h compared to untreated negative control cells. The data represent the mean (n = 3) ± SD.

The antileishmanial effects of free ATRA and ATRA-loaded SLNs on *L. infantum* promastigotes were evaluated using the Alamar Blue assay at 25, 50, and 75 μM for 24, 48, and 72 h (Figure 2). As shown in Figure 2A, free ATRA did not produce a marked reduction in promastigote viability at any concentration or time point. Viability values remained close to or above negative control values across the entire experimental period, ranging from approximately 95% to 120%, indicating that free ATRA at ≤75 μM does not exert a measurable inhibitory effect on extracellular promastigotes.

A comparable outcome was obtained with the ATRA-loaded SLNs (Figure 2B). Across all tested concentrations and time points, promastigote viability fluctuated within roughly the 95%–150% range, and none of the doses produced a meaningful reduction in metabolic activity. These results indicate that encapsulating ATRA into SLNs does not enhance its direct antileishmanial activity on promastigotes at the concentration range evaluated. Taken together, both free ATRA and ATRA-loaded SLNs exhibited minimal direct activity against *L. infantum* promastigotes at concentrations up to 75 μM.

#### 3.2.2. Cytotoxicity of free ATRA and ATRA-loaded SLNs on RAW264.7 macrophages

**Figure 3:**
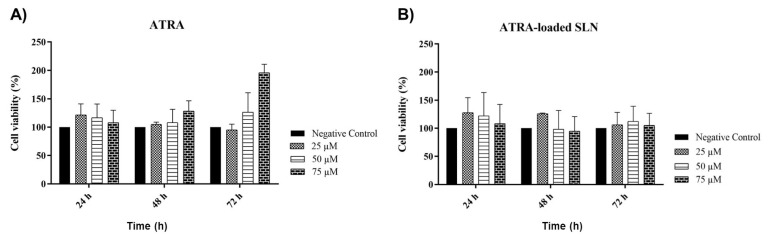
Cytotoxicity of (A) free ATRA and (B) ATRA-loaded SLNs on macrophages. The viability analysis of RAW264.7 macrophages treated with (A) free ATRA (in ethanol) and (B) ATRA-loaded SLNs was investigated at various concentrations (0–75 μM) for 24, 48, and 72 h compared to untreated negative control cells. The data represent the mean (n = 3) ± SD.

Following the antileishmanial evaluation against *L. infantum* promastigotes, the cytotoxicity of free ATRA and its SLN formulation was assessed separately on noninfected RAW 264.7 macrophages for 24, 48, and 72 h using the Alamar Blue assay (Figure 3). As shown in Figure 3A, treatment with free ATRA at concentrations of 0–75 μM did not induce any cytotoxic response in healthy macrophages. At all time points, cell viability remained comparable to or slightly above that of the negative control group, with values ranging between approximately 100% and 121% at 24 h and remaining above 95% even after 72 h of incubation. The results show that free ATRA does not have any harmful effect on RAW 264.7 macrophages at the concentrations used in the study (25–75 μM).

When ATRA was incorporated into the SLN formulation, the system exhibited biocompatibility consistent with the free compound. Cell viability remained stable at all tested concentrations up to 75 μM, with no significant reduction observed at any of the time points evaluated (24, 48, or 72 h) (Figure 3B). Moreover, treatment with the SLN formulation indicated that the nanoparticles were well tolerated by the macrophages, similar to free ATRA. The increase in the percentage of macrophages was most noticeable at 25 and 50 μM, where the cells were around 128% viable after 24 and 48 h. After 72 h, the values were still around 105% and 110%, respectively. Likewise, at 75 μM, the effect maintains stable macrophage viability, suggesting increased metabolic activity rather than a toxic response.

The results show that neither the free form of ATRA nor the SLN-encapsulated formulation affects the survival of macrophages. The antileishmanial activity observed in noninfected macrophages, therefore, does not appear to stem from host cell damage. Instead, the data point to ATRA, particularly when delivered through SLNs, as a well-tolerated approach for influencing the intracellular amastigotes while maintaining macrophage integrity.

#### 3.2.3. Antileishmanial efficacy of free ATRA and ATRA-loaded SLNs on *Leishmania*-infected macrophages

**Figure 4:**
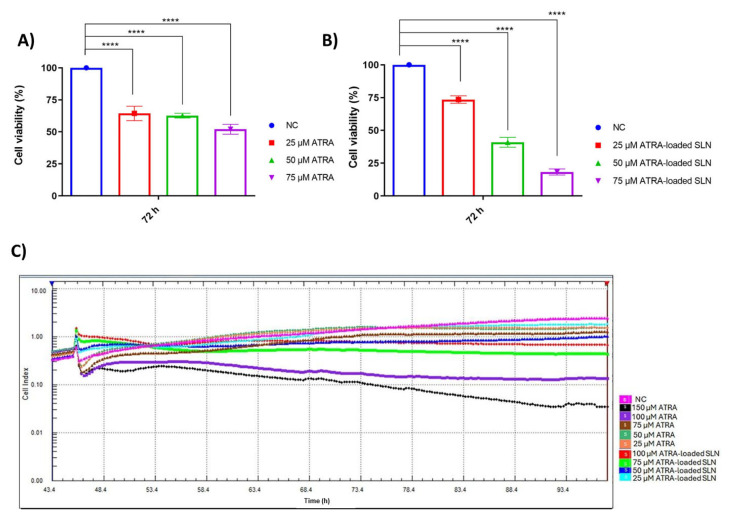
Cell index-derived response of *L. infantum*-infected macrophages following 72 h of treatment with (A) free ATRA and (B) ATRA-loaded SLNs at different concentrations (0–75 μM). (C) Real-time cell index curves obtained using the xCELLigence RTCA system. Data are presented as mean (n = 3) ± SD.

Following 72 h of incubation, free ATRA caused a clear reduction in the viability of macrophages infected with *Leishmania* parasites. At concentrations of 25, 50, and 75 μM, cell viability dropped to 64.43%, 62.71%, and 52.02%, respectively (p ≤ 0.0001), indicating a consistent inhibitory effect across these doses (Figure 4A).

In contrast, when ATRA was delivered within the SLN formulation, the intracellular response was noticeably stronger. After 72 h, viability levels were 73.54%, 40.94%, and 18.26% for 25, 50, and 75 μM, respectively (p ≤ 0.0001) (Figures 4B and 4C). The reduction in viability showed a clear dose-dependent pattern, particularly between 50 and 75 μM. This suggests that encapsulation enhances the ability of ATRA to reach the interior of infected macrophages and strengthens host-mediated antileishmanial activity.

When the two forms were compared at equal concentrations, the SLN-loaded ATRA consistently produced a greater reduction in viability than free ATRA did. This difference was most pronounced at 50 and 75 μM, indicating that nanoparticle-based delivery is more effective at *Leishmania*-infected macrophages.

Taken together, the findings demonstrate that incorporating ATRA into SLNs markedly improves its antileishmanial efficacy. The stronger response observed at intermediate doses (50–75 μM), combined with the absence of toxicity in noninfected macrophages, supports the idea that SLN-mediated delivery offers a more targeted and potentially safer strategy for treating intracellular amastigotes.

## 4. Discussion

In the present study, we evaluated the antileishmanial activity of ATRA in both its free form and when encapsulated within SLN nanoparticles. The therapeutic effects were assessed using extracellular parasites as well as infected and noninfected macrophages to determine both antiparasitic efficacy and host cell compatibility. The results demonstrate that SLN-mediated delivery significantly enhances the intracellular efficacy of ATRA while maintaining macrophage viability. These findings support and extend the emerging view that retinoids, particularly ATRA, function primarily as host-directed immunomodulators rather than direct parasiticidal agents in leishmaniasis. Within this framework, the physicochemical properties of the SLN formulation play an important role in determining the intracellular delivery and availability of ATRA.

The encapsulation efficiency of ATRA within the SLN system was 19.6%. Although this value may appear relatively low compared with those of some lipid-based delivery systems, it is consistent with previously reported values for lipophilic compounds incorporated into tripalmitin-based SLNs (Islek et al., 2024). In earlier studies, the encapsulation amount of lipophilic drugs within similar tripalmitin-based SLN matrices was reported to range between 0.02 and 0.1, indicating that the encapsulation levels observed in the present formulation fall within the expected range for this lipid system (Ucisik et al., 2013a, 2013b; Bolat et al., 2020). Such encapsulation levels have been attributed to the limited loading capacity of the solid lipid matrix, where the incorporation of increasing amounts of lipophilic molecules eventually leads to a saturation plateau within the lipid core (Islek et al., 2024). This phenomenon reflects the intrinsic structural characteristics of the lipid matrix rather than a limitation of the formulation strategy. Importantly, despite the moderate encapsulation efficiency, the resulting ATRA concentration within the SLN formulation remained sufficient to achieve the therapeutic concentration range required for the in vitro macrophage infection model used in the present study.

Consistent with the concept, free ATRA displayed only modest or negligible effects on extracellular promastigotes at concentrations up to 75 μM, whereas a pronounced reduction in viability was observed in *Leishmania*-infected macrophages, especially at 50–75 μM. This disconnect between promastigote and intracellular amastigote models suggests that ATRA’s primary antileishmanial action is not direct parasite killing in the extracellular phase but rather relies on changes induced within the host cell environment. A similar concept has been supported by clinical and ex vivo data in visceral leishmaniasis: Maciel et al. (2014) showed that vitamin A metabolites, including ATRA, modulate IL-10, TGF-β, and IL-17 production in T cells and monocytes from VL patients and endemic controls, with effects that depend strongly on the immunological context and the presence of leishmanial antigens. Our findings support the concept that ATRA plays a complex immunoregulatory role rather than functioning as a classical cytotoxic agent. Instead of directly targeting the parasites, ATRA appears to modulate host immune responses at the intracellular interface between the parasite and the macrophage, where the infection is established.

The enhanced activity of ATRA-loaded SLNs in infected macrophages reinforces this host-dependent interpretation. At equivalent or even lower concentrations than free ATRA, the SLN formulation produced substantially greater reductions in the viability of *L. infantum*-infected macrophages, particularly at 50 and 75 μM, while sparing noninfected RAW 264.7 cells. Nanoparticles in the ~120 nm range with a negative zeta potential, as in our formulation, are efficiently internalized by macrophages and can accumulate within endosomal and phagolysosomal compartments, where the intracellular amastigote resides. This is consistent with prior work showing that retinoic acid supplementation in *L. donovani*-infected macrophages restores cholesterol homeostasis via upregulation of NPC1/NPC2, thereby limiting parasite survival, and that RA deficiency favors parasite persistence (Prakash et al., 2022). Together, these findings support the idea that ATRA-loaded SLN systems potentiate host-directed mechanisms, such as metabolic reprogramming or altered endolysosomal function, that render the intracellular niche less permissive for *Leishmania*.

Interestingly, not all studies agree on whether ATRA is beneficial for host resistance in leishmaniasis. Vellozo et al. (2017) reported that ATRA can drive macrophages from an M1-like to an M2-like phenotype and impair macrophage-mediated immunity to *L. major* in murine models, leading to reduced control of infection. This appears, at first glance, to contrast with our observation that ATRA, particularly in SLN form, enhances killing of *L. infantum* within macrophages. Several factors may help reconcile these differences: species-specific biology (*L. major* vs. *L. infantum/donovani*), disease form (cutaneous vs. visceral), host background, ATRA dose and timing, and, critically, the use of a nanoformulation that alters drug distribution and kinetics. Moreover, human studies in VL have shown that ATRA can exert differential immunomodulatory effects depending on the cellular context. In healthy cells, ATRA has been reported to increase regulatory cytokine production, whereas in the presence of *Leishmania* antigens, it can limit IL-10 production in cells derived from VL patients. These observations further support the concept that ATRA functions as a context-dependent immunomodulator rather than a uniformly suppressive agent (Maciel et al., 2014).

Our results also resonate with broader work on retinoids and leishmaniasis that frame these molecules as components of host-directed therapeutic strategies. In canine leishmaniasis, in vitro supplementation of leukocyte cultures with ATRA, vitamin D3, and zinc has been reported to modulate nitric oxide, reactive oxygen species, and cytokine production, altering the leishmanicidal activity of immune cells (Hernandez et al., 2021). More recently, network- and systems-based analyses have emphasized macrophages and monocytes as central nodes for host-directed antileishmanial interventions, including retinoid pathways (Martinez-Hernandez et al., 2021). Our data, showing strong intracellular activity of ATRA-loaded SLNs in the absence of toxicity to noninfected macrophages, are fully compatible with this host-directed therapy (HDT) framework, and suggest that ATRA might be repositioned as a component of HDT regimens rather than as a stand-alone antiparasitic.

From a nanomedicine perspective, our findings are also in line with previous work demonstrating that nanoencapsulated retinoic acid can be used safely as an immunomodulatory adjuvant. Bezerra et al. reported that RA-loaded SLNs acted as a well-tolerated tolerogenic adjuvant in vaccination strategies against leishmaniasis, promoting regulatory responses without overt toxicity (Bezerra et al., 2019). While the immunological goal in that context (tolerogenic adjuvanticity) differs from the therapeutic intent of our study (host-mediated antileishmanial activity), both sets of data converge on a key point: SLN-based RA formulations can be administered safely and can shape immune responses in a controlled manner. Our findings further support the concept of host-directed therapy by demonstrating that the ATRA-loaded SLN formulation enhances the control of amastigotes within macrophages while preserving host cell viability. This suggests that nanoparticle-mediated delivery enables ATRA to modulate macrophage-mediated antiparasitic responses rather than exerting direct parasiticidal toxicity. Collectively, these results highlight the potential of the ATRA-SLN system as a host-mediated therapeutic strategy against intracellular *Leishmania* infection.

In summary, our study supports the idea that ATRA, especially when delivered via SLNs, works as a host-directed modulator. This means it helps macrophages control the parasites inside them. It does not just directly fight the parasites. The drug is effective against disease inside the body, and it does not harm the cells that are not infected. This places ATRA-SLNs into the group of medicines that target the immune system to fight leishmaniasis (Mahor et al., 2023). In the future, we should look more closely at the exact mechanisms involved, like how macrophages change, fat metabolism, and checkpoint or cytokine networks. We should also study how ATRA-SLNs work in live models of VL, either on their own or with other standard antileishmanial drugs. Such studies will be essential to determine whether the promising in vitro profile observed here can be translated into a clinically relevant, host-centered therapeutic strategy.

## 5. Conclusion

In the present study, it is demonstrated that SLNs significantly enhance the host-mediated antileishmanial activity of ATRA against *L. infantum*-infected macrophages, while maintaining a favorable safety profile in healthy cells. The limited effect of ATRA on extracellular promastigotes, contrasted with its strong activity in infected macrophages, underscores a host-dependent mechanism rather than a direct antiparasitic action. SLN encapsulation further potentiates this mechanism, likely through improved macrophage uptake and sustained intracellular drug release. Together, these findings position ATRA-loaded SLNs as a viable candidate for host-directed therapy in visceral leishmaniasis. Future investigations should explore mechanistic pathways underlying this host–parasite modulation, evaluate activity against intracellular amastigotes, and assess in vivo therapeutic performance.
